# Potential Host-Directed Mechanisms of *Houttuynia cordata* in *Bovine Mycoplasma bovis* Pneumonia: A Network Pharmacology and Molecular Docking Study

**DOI:** 10.3390/vetsci13070658

**Published:** 2026-07-07

**Authors:** Meihe Zhao, Tingyu Li, Liyin Du, Qinghua Deng, Jingdong Mao, Zhenwei Jia, Yuming Zhang

**Affiliations:** 1 College of Animal Science and Technology, Inner Mongolia Minzu University, Tongliao 028000, China; 15612481858@163.com (M.Z.); 18035361054@163.com (T.L.);; 2 Inner Mongolia Engineering Technology Research Center for Prevention and Control of Beef Cattle Diseases, Tongliao 028000, China; 3 Beef Cattle Industry School of Inner Mongolia, Tongliao 028000, China

**Keywords:** *Houttuynia cordata*, *bovine Mycoplasma bovis pneumonia*, host-directed regulation, network pharmacology, molecular docking, flavonoids, inflammation, immune response

## Abstract

Bovine *Mycoplasma bovis* pneumonia (MBP) is an important component of bovine respiratory disease, and its management is complicated by persistent infection and antimicrobial stewardship concerns. *Houttuynia cordata* Thunb. has reported anti-inflammatory and immunomodulatory activities, but its potential host-directed mechanisms in MBP remain unclear. This in silico study used network pharmacology and molecular docking to identify candidate compounds, common drug–disease targets, enriched biological functions, and predicted ligand–target interactions. A total of 145 putative targets of *H. cordata* and 474 MBP-associated disease targets were obtained from TCMSP, GeneCards, OMIM, and CTD, yielding 43 common drug–disease targets. Dual-confidence STRING analysis, cytoHubba ranking, and MCODE module analysis prioritized TNF, IL6, IL1B, PTGS2, PPARG, IFNG, CASP3, and MMP9 as candidate core targets. Gene Ontology (GO) and Kyoto Encyclopedia of Genes and Genomes (KEGG) enrichment indicated convergence on cytokine-mediated signaling, inflammatory response, immune regulation, oxidative stress, IL-17 signaling, and TNF signaling. Molecular docking suggested favorable predicted interactions for quercitrin–PTGS2, quercetin–TNF, quercetin–IL6, and quercitrin–CASP3. These computational findings suggest that *H. cordata* may be associated with host inflammatory and immune-response modulation in MBP, mainly through flavonoid-related interactions with inflammation- and apoptosis-related targets. Further bovine-specific experimental validation is required before biological activity or practical application can be inferred.

## Simple Summary

Pneumonia caused by *Mycoplasma bovis* is difficult to manage in cattle because the infection can persist, injure lung tissue, and reduce the effectiveness of antimicrobial control. *Houttuynia cordata* is a medicinal and edible plant with reported anti-inflammatory and immune-regulating activities, but its possible role in this disease remains unclear. This study used computer-based network analysis and molecular docking to explore whether compounds from *H. cordata* may interact with host molecules involved in inflammation, immune imbalance, cell injury, and lung damage during bovine *M. bovis* pneumonia. The results suggested that several flavonoids, especially quercetin and quercitrin, may interact with host targets linked to inflammatory signaling and cell death. These findings do not show that *H. cordata* treats or prevents *M. bovis* pneumonia, and they should not be interpreted as a replacement for veterinary diagnosis or antimicrobial therapy. Instead, this study provides preliminary mechanistic clues for future bovine cell and animal studies evaluating whether *H. cordata* could support respiratory health as an auxiliary natural resource in cattle.

## 1. Introduction

*Mycoplasma bovis* is an important pathogen involved in the bovine respiratory disease complex and can cause bovine *Mycoplasma bovis* pneumonia (MBP). In addition to respiratory disease, *M. bovis* is associated with arthritis, otitis media, mastitis, and other clinical disorders in cattle [1,2]. MBP is commonly observed in calves and feedlot cattle, and affected animals may present with fever, coughing, dyspnea, and varying degrees of pulmonary lesions [1,3]. As part of the bovine respiratory disease complex, *M. bovis* infection can occur together with other viral or bacterial pathogens, thereby aggravating respiratory inflammation and disease progression [3]. Beyond direct pathogen-associated injury, *M. bovis* infection is characterized by chronicity, persistence, and immune-evasion capacity, which may contribute to prolonged inflammatory responses, immune dysregulation, and recurrent pulmonary tissue damage [2,4]. These pathological processes impair animal health, welfare, and productivity and can cause substantial economic losses [1,2]. Therefore, MBP represents a clinically and economically relevant disease context for exploring host-response regulatory mechanisms in cattle.

The absence of a cell wall makes *M. bovis* intrinsically insensitive to β-lactam antimicrobials that target cell-wall synthesis [5]. Antimicrobial treatment remains one of the main approaches for managing *M. bovis*-associated diseases, with macrolides, tetracyclines, and fluoroquinolones among the commonly used drug classes [5,6]. Declining antimicrobial susceptibility and increasing resistance in *M. bovis* have raised concerns regarding treatment efficacy and antimicrobial stewardship [5,6,7]. Since MBP involves not only pathogen persistence but also host inflammatory amplification, immune imbalance, and pulmonary tissue injury, auxiliary approaches that focus on host-response modulation may be valuable for future respiratory health management in cattle. Such approaches should be considered complementary to, rather than replacements for, veterinary diagnosis and antimicrobial therapy.

Plant-derived extracts and bioactive compounds have received increasing attention in animal health management and antimicrobial-reduction strategies because of their diverse biological activities and potential to regulate host inflammation and immunity [8,9]. *Houttuynia cordata* Thunb. is a traditional medicinal plant and medicinal-edible resource widely used in Asian countries [8,10]. Phytochemical studies have shown that *H. cordata* contains multiple classes of constituents, including volatile oils, flavonoids, polysaccharides, alkaloids, and organic acids, which have been associated with anti-inflammatory, antioxidant, immunomodulatory, and antimicrobial activities in different experimental contexts [8,10]. Among these constituents, flavonoids such as quercetin, quercitrin, and kaempferol are closely related to inflammatory regulation and antioxidant responses; volatile constituents such as decanoyl acetaldehyde and 2-undecanone contribute to the characteristic chemical profile and source specificity of *H. cordata*; and polysaccharides are more frequently associated with immune modulation, including macrophage activation, cytokine regulation, and lymphocyte-related responses [8,9,10]. Recent animal-production-oriented reviews have also discussed *H. cordata* extract as a natural resource with antimicrobial, anti-inflammatory, antioxidant, and immune-regulating potential, suggesting possible value in supporting disease resistance, reducing inflammatory stress, and limiting excessive dependence on antimicrobials [9]. However, its potential relevance to MBP should be interpreted primarily from the perspective of host inflammatory and immune regulation rather than direct pathogen elimination.

Existing studies provide experimental support for the anti-inflammatory and immunomodulatory properties of *H. cordata*. In LPS-induced RAW264.7 macrophages and carrageenan-induced paw edema models, *H. cordata* fermentation products have been reported to reduce inflammatory responses, indicating activity in both cellular and animal inflammation models [11]. Other studies have shown that *H. cordata* extracts can suppress pro-inflammatory mediators in LPS-stimulated macrophages and lung-related inflammatory models, including IL-1β, IL-6, TNF-α, nitric oxide, NF-κB activation, and ERK phosphorylation [12,13]. In addition to its anti-inflammatory effects, *H. cordata* preparations and polysaccharide-related components have been associated with immune modulation, including effects on phagocytic activity, antibody production, macrophage polarization, cytokine regulation, and T-cell-related immune responses [9,13,14]. The close association of MBP with persistent inflammatory responses, cytokine-network imbalance, immune dysregulation, oxidative stress, apoptosis, and pulmonary tissue injury provides a biological basis for hypothesizing that *H. cordata* may be associated with MBP-related host-response modulation through inflammation- and immunity-related regulatory networks [1,4,12,13,14].

Despite these findings, existing evidence does not fully explain how the complex constituents of *H. cordata* correspond to MBP-related host targets and signaling pathways. The multiple classes of bioactive compounds in *H. cordata* may interact with widely distributed molecular targets, but the key active compounds, core host targets, and major pathway networks associated with MBP remain unclear [8,10]. Network pharmacology provides a systems-level strategy for exploring the multi-component, multi-target, and multi-pathway characteristics of medicinal plants from a compound–target–pathway–disease perspective, making it suitable for generating mechanistic hypotheses for complex medicinal-edible plant resources [15]. Molecular docking can further assess predicted ligand–target interactions at the structural level and provide supplementary support for network-based predictions [16]. In the present in silico study, network pharmacology was used to screen potential active compounds and putative targets of *H. cordata*, and these targets were integrated with MBP-related disease targets. Protein–protein interaction (PPI) network construction, core-target prioritization, Gene Ontology (GO) and Kyoto Encyclopedia of Genes and Genomes (KEGG) enrichment analysis, and molecular docking were performed to investigate potential host-directed mechanisms by which *H. cordata* may be associated with MBP-related inflammatory and immune processes. This study aims to provide candidate compounds, core targets, and pathway-level clues for future bovine-specific validation of *H. cordata* as a potential host-response-modulating auxiliary resource in the context of MBP.

## 2. Materials and Methods

### 2.1. Study Design and Reporting Scope

This was an in silico hypothesis-generating study based on public databases, network pharmacology, topological network analysis, functional enrichment analysis, and molecular docking. No live animals, clinical samples, animal tissues, or additional laboratory experiments were used. The analyses were designed to identify candidate compounds, host targets, and signaling pathways potentially associated with the regulation of MBP-related inflammatory and immune processes by *Houttuynia cordata*. Therefore, all biological interpretations were framed as computational predictions rather than direct evidence of therapeutic efficacy, target regulation, or direct antimycoplasmal activity.

### 2.2. Databases and Software Platforms

The databases and online platforms used in this study included the Traditional Chinese Medicine Systems Pharmacology Database and Analysis Platform (TCMSP) [17], GeneCards [18], OMIM [19], CTD [20], UniProt [21], jvenn [22], STRING [23], PubChem [24], RCSB PDB [25], and an online bioinformatics platform for visualization. Database searches and key information, including compound information, disease-related targets, protein annotations, protein–protein interaction networks, ligand structures, and receptor structures, were checked or rechecked on 23 June 2026. Network construction and topological analyses were performed using Cytoscape (version 3.10.4; Cytoscape Consortium, San Diego, CA, USA) [26] together with the NetworkAnalyzer, cytoHubba [27], and MCODE [28] plugins. Gene Ontology (GO) and Kyoto Encyclopedia of Genes and Genomes (KEGG) enrichment analyses were conducted using R packages, including clusterProfiler [29], org.Bt.eg.db, enrichplot [30], and ggplot2 [31]. Ligand structure optimization was performed using Chem3D (version 22.0; PerkinElmer Informatics, Waltham, MA, USA). Receptor and ligand preparation was performed using AutoDockTools (version 1.5.7; The Scripps Research Institute, La Jolla, CA, USA) [32], molecular docking was conducted using AutoDock Vina (version 1.1.2; The Scripps Research Institute, La Jolla, CA, USA) [33], and docking conformations were visualized using PyMOL (version 3.0; Schrödinger, LLC, New York, NY, USA).

### 2.3. Screening of Candidate and Representative Compounds of Houttuynia cordata

Chemical constituents and related target information of *H. cordata* were retrieved from TCMSP using “yuxingcao” and “*Houttuynia cordata*” as search terms. Oral bioavailability (OB) ≥ 30% and drug-likeness (DL) ≥ 0.18 were used as preliminary screening criteria. Compounds meeting these criteria were included as candidate active compounds. Representative compounds with clear literature support but limited database annotation were manually verified based on the references cited in this manuscript to improve the rationality and completeness of compound screening without adding new experimental information. Characteristic constituents of *H. cordata*, especially volatile compounds with source-specific relevance, were retained when supported by published evidence, even if their ADME parameters did not fully meet the conventional TCMSP thresholds. Therefore, the compound set included both database-screened candidate compounds and literature-curated representative constituents.

### 2.4. Acquisition and Standardization of Putative Targets of Houttuynia cordata

The predicted targets corresponding to the candidate active compounds of *H. cordata* were collected and deduplicated. UniProt was used to annotate and standardize protein names, which were converted into official gene symbols. For targets with aliases, outdated names, or inconsistent nomenclature across databases, manual curation was performed to generate a standardized putative target set of *H. cordata*. The deduplicated and standardized target set was used for subsequent common-target screening and network analysis.

### 2.5. Acquisition and Standardization of MBP-Related Candidate Disease Targets

Disease-related genes of bovine *Mycoplasma bovis* pneumonia were retrieved from GeneCards, OMIM, and CTD using “*Mycoplasma bovis* pneumonia” and “bovine mycoplasma pneumonia” as the main search terms. Targets obtained from different databases were integrated, deduplicated, and manually curated. Gene names, aliases, and abbreviations were standardized to construct a candidate disease-target set for MBP.

GeneCards, OMIM, and CTD are mainly constructed from human-centered or general biomedical knowledge, whereas systematic bovine-specific disease-target databases for bovine *Mycoplasma bovis* pneumonia are limited. These databases were therefore used as initial knowledge sources for collecting candidate targets related to MBP-associated pathological processes, including inflammatory response, cytokine signaling, immune regulation, and pulmonary tissue injury. These pathological processes are partially conserved among mammals and can provide useful references for mechanistic prediction in MBP, although cross-species annotation bias cannot be completely avoided. To improve relevance to the bovine disease context, disease-target names were standardized, and the species was restricted to *Bos taurus* during STRING protein–protein interaction (PPI) construction. During STRING species mapping, targets that could not be recognized or mapped to the bovine protein-interaction network were excluded from PPI analysis. This approach was used to reduce, as far as possible, the cross-species bias introduced by the direct use of human-centered disease knowledge databases.

### 2.6. Screening of Common Drug–Disease Targets

The standardized putative targets of *H. cordata* and the candidate disease targets of MBP were imported into jvenn for intersection analysis. The overlapping targets were defined as common drug–disease targets and were used for subsequent PPI network construction, topological analysis, functional enrichment analysis, and molecular docking target selection. A Venn diagram was generated to visualize the overlap between the *H. cordata* target set and the MBP-related disease-target set.

### 2.7. Construction of the Compound–Target Network and Screening of Key Compounds

Candidate active compounds of H. cordata and their corresponding common targets were imported into Cytoscape to construct a compound–target network. Nodes represented active compounds or targets, and edges represented predicted compound–target associations. NetworkAnalyzer was used to calculate topological parameters, especially Degree, to evaluate the relative network importance of compounds and targets. Compounds with higher Degree values were considered to have broader predicted target-interaction potential and were preferentially selected as candidate ligands for molecular docking. These network-based rankings were interpreted as topological prioritization rather than direct evidence of pharmacological efficacy.

### 2.8. PPI Network Construction, Topological Analysis, and Module Screening

The common targets between *H. cordata* and MBP were imported into STRING to construct PPI networks, with the species restricted to *Bos taurus*. A dual-confidence strategy was used to balance network coverage and interaction reliability. First, a global interaction network was constructed under the medium-confidence threshold, with the minimum required interaction score set to 0.400, to retain potential regulatory relationships. A high-confidence network was then constructed under the high-confidence threshold, with the minimum required interaction score set to 0.700, to extract a more stringent interaction framework. No additional interactors were added.

Topological analyses of the STRING 0.4 and STRING 0.7 PPI networks were performed using NetworkAnalyzer in Cytoscape. Degree, Betweenness Centrality, and Closeness Centrality were calculated to evaluate node centrality. To reduce bias caused by a single topological metric, cytoHubba was used for multi-algorithm hub-target screening. Four algorithms, including MCC, Degree, Betweenness, and Closeness, were used to rank the nodes, and the top 10 genes from each algorithm were extracted. High-confidence core targets were selected based on occurrence frequency, cross-threshold reproducibility, and ranking stability.

MCODE was used to identify densely connected regions in the PPI networks. The parameters were set as follows: Degree Cutoff = 2, Node Score Cutoff = 0.2, K-Core = 2, and Max Depth = 100. Key functional modules were selected based on module scores and gene composition. Genes repeatedly appearing in key modules and consistent with core-target screening results were considered to have higher network-level mechanistic relevance. The hub-target and module-screening results were interpreted as network-topological prioritization rather than experimental confirmation of biological function.

### 2.9. GO and KEGG Enrichment Analyses

To clarify the potential biological functions of common and core targets, GO functional enrichment analysis and KEGG pathway enrichment analysis were performed using R. Gene symbols were converted into Entrez IDs, and the species background was set as *Bos taurus*. GO enrichment analysis included Biological Process (BP), Cellular Component (CC), and Molecular Function (MF). For KEGG enrichment analysis, the organism code was set as bta. Multiple testing correction was performed using the Benjamini–Hochberg method, and terms with *p* < 0.05 or adjusted *p* value < 0.05 were considered statistically significant. Significantly enriched terms were visualized using bubble plots and bar plots.

### 2.10. Integrated Compound–Target–Pathway Analysis

Based on key active compounds, prioritized core targets, and significantly enriched pathways, an integrated compound–target–pathway interpretation was conducted to clarify the potential mechanistic axis of *H. cordata* in MBP. This integrated interpretation was used to support the proposed host-directed mechanism and the integrated evidence matrix provided in Supplementary Table S1.

### 2.11. Selection of Ligand–Target Pairs for Molecular Docking

Exhaustive docking of all active compounds against all core targets was not performed. Representative active compounds and key targets were selected based on network topological features, pharmacological relevance, docking feasibility, and the functional categories of core targets. The final ligand–target pairs included quercetin–TNF, quercetin–IL6, quercetin–PPARG, kaempferol–IL1B, kaempferol–CASP3, quercitrin–PTGS2, quercitrin–CASP3, decanoyl acetaldehyde–MMP9, decanoyl acetaldehyde–PTGS2, and 2-undecanone–PPARG. These pairs covered major pathological processes implicated in MBP-associated host responses, including inflammatory cytokine regulation, immune modulation, apoptosis, and tissue injury. Molecular docking was therefore used to provide structural-level clues for representative compound–target interactions predicted by network pharmacology, rather than to validate biological regulation.

### 2.12. Molecular Docking Procedure 

The three-dimensional structures of candidate active compounds were downloaded from PubChem in SDF or MOL2 format. Ligand structures were energy-minimized using Chem3D and converted into PDBQT format using AutoDockTools. Crystal structures of core target proteins were downloaded from the RCSB PDB database. Candidate receptor proteins included TNF, IL6, PPARG, IL1B, CASP3, PTGS2, and MMP9. Receptor structures were preprocessed using PyMOL and AutoDockTools, including removal of water molecules, deletion of original ligands, addition of polar hydrogens, calculation of Gasteiger charges, and conversion into PDBQT format.

Semi-flexible molecular docking was performed using AutoDock Vina, with receptor proteins treated as rigid and ligands treated as flexible. The grid box was set to cover the co-crystallized ligand-binding region, known functional pocket, or reported functional/interface-associated region of each receptor. For proteins without a clearly defined co-crystallized small-molecule ligand, docking regions were defined based on reported structural features or functional regions. Binding energy (kcal/mol) was used as the evaluation index, and lower binding energy was considered to indicate stronger predicted ligand–receptor binding under the present docking conditions. Binding energy < −5.0 kcal/mol was used as an empirical indicator of possible binding potential in this docking analysis. Molecular docking results were interpreted as predicted structural interactions and not as direct evidence of target regulation or biological activity. Detailed ligand PubChem CIDs, receptor PDB IDs, structure-selection rationale, docking-region definitions, and docking energies are provided in Supplementary Tables S2 and S5.

To summarize the robustness of the prioritized core targets, evidence from common target screening, dual-threshold STRING analysis, cytoHubba ranking, MCODE module membership, GO and KEGG enrichment results, molecular docking, and relevant literature was compiled into an integrated evidence matrix. This matrix was used only as a descriptive summary of multi-layer support and was not regarded as experimental validation.

## 3. Results

### 3.1. Representative Compounds and Putative Targets of Houttuynia cordata

Chemical constituents of *H. cordata* were retrieved from TCMSP using oral bioavailability (OB) ≥ 30% and drug-likeness (DL) ≥ 0.18 as preliminary screening criteria. Published studies cited in this manuscript were used to supplement and manually verify representative constituents frequently reported in *H. cordata*, especially those associated with anti-inflammatory, immunomodulatory, or characteristic volatile-component properties. The representative compounds highlighted in this study included TCMSP-screened flavonoids and literature-curated characteristic volatile constituents, namely quercetin, kaempferol, quercitrin, decanoyl acetaldehyde, and 2-undecanone (Table 1). Quercetin, kaempferol, and quercitrin are flavonoids or flavonoid glycosides, whereas decanoyl acetaldehyde and 2-undecanone are characteristic volatile constituents of *H. cordata*. After integration, deduplication, and standardization of targets corresponding to the screened and literature-curated compounds, 145 putative targets of *H. cordata* were obtained for subsequent drug–disease target intersection and network analysis.

### 3.2. MBP-Related Disease Targets and Common Drug–Disease Targets

**Figure 1:**
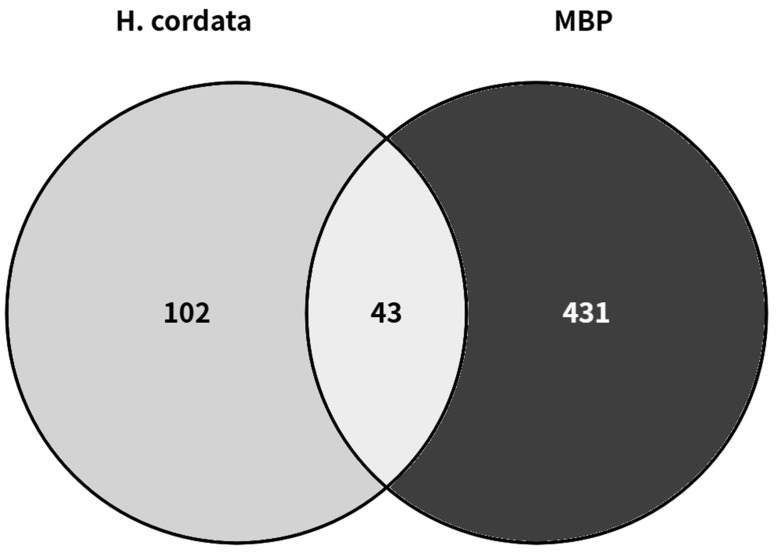
Venn diagram showing the common targets between *Houttuynia cordata* and bovine *Mycoplasma bovis* pneumonia. A total of 145 putative targets of *H. cordata* and 474 MBP-related disease targets were intersected using jvenn, yielding 43 common drug–disease targets. The *H. cordata*-only and MBP-only regions contained 102 and 431 targets, respectively. MBP, bovine *Mycoplasma bovis* pneumonia.

To construct the MBP-related disease-target set, GeneCards, OMIM, and CTD were searched using “*Mycoplasma bovis* pneumonia” and “bovine mycoplasma pneumonia” as the main keywords. Targets obtained from different databases were integrated, deduplicated, standardized, and manually curated. After this process, 474 MBP-related candidate disease targets were obtained. For intersection analysis, 145 putative targets of *Houttuynia cordata* and 474 MBP-related candidate disease targets were imported into jvenn, yielding 43 common drug–disease targets (Figure 1). These common targets included cytokines, chemokines, inflammatory signaling molecules, oxidative-stress-related genes, apoptosis-associated genes, immune-regulatory genes, and tissue-remodeling-related genes, represented by TNF, IL6, IL1B, PTGS2, PPARG, IFNG, CASP3, CXCL8, IL10, and MMP9 (Table 2). These common targets were used for subsequent protein–protein interaction (PPI) network construction, core-target screening, Gene Ontology (GO) and Kyoto Encyclopedia of Genes and Genomes (KEGG) enrichment analyses, and molecular docking analysis.

### 3.3. PPI Network Construction of Common Targets

The 43 common targets shared by *Houttuynia cordata* and MBP were imported into the STRING database, with the species restricted to *Bos taurus*, to construct protein–protein interaction (PPI) networks. A total of 42 nodes were retained in the STRING networks, indicating that one common target was not effectively mapped to the bovine STRING interaction dataset under the current retrieval conditions.

**Figure 2:**
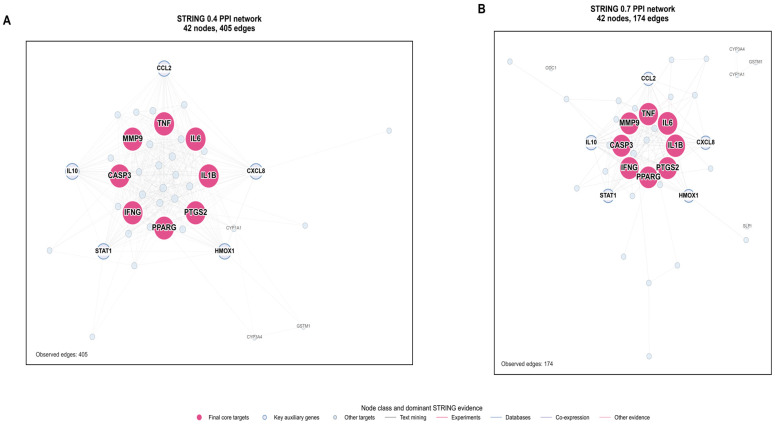
Protein–protein interaction (PPI) networks of common targets between *Houttuynia cordata* and bovine *Mycoplasma bovis* pneumonia. The networks were constructed using STRING with the species restricted to *Bos taurus* and no additional interactors. (**A**) Medium-confidence network, interaction score ≥ 0.400, containing 42 nodes and 405 edges. (**B**) High-confidence network, interaction score ≥ 0.700, containing 42 nodes and 174 edges. Pink nodes indicate prioritized final core targets, blue-outlined nodes indicate key auxiliary genes, and light-gray nodes indicate other common targets.

Under the medium-confidence threshold (minimum required interaction score = 0.400), the PPI network contained 42 nodes and 405 edges, with an average node degree of 19.3, an average local clustering coefficient of 0.796, an expected number of edges of 67, and a PPI enrichment *p* < 1.0 × 10^−16^ (Figure 2A). These results indicate that the common targets were more interconnected than expected by chance, suggesting potential functional associations among the predicted *H. cordata*–MBP common targets.

Under the high-confidence threshold (minimum required interaction score = 0.700), the PPI network contained 42 nodes and 174 edges, with an average node degree of 8.29, an average local clustering coefficient of 0.654, an expected number of edges of 20, and a PPI enrichment *p* < 1.0 × 10^−16^ (Figure 2B). Compared with the medium-confidence network, the high-confidence network retained fewer interactions and provided a more stringent interaction framework for subsequent core-target screening.

The medium-confidence network was used to characterize the global interaction landscape of the common targets, whereas the high-confidence network was used to evaluate a more stringent subset of predicted interactions. The significant PPI enrichment observed under both thresholds suggests that the common targets of *H. cordata* and MBP were not randomly distributed in the bovine protein-interaction background, but were clustered within functionally associated host-response-related networks.

### 3.4. Multi-Algorithm cytoHubba Screening in the STRING 0.4 Network

**Figure 3:**
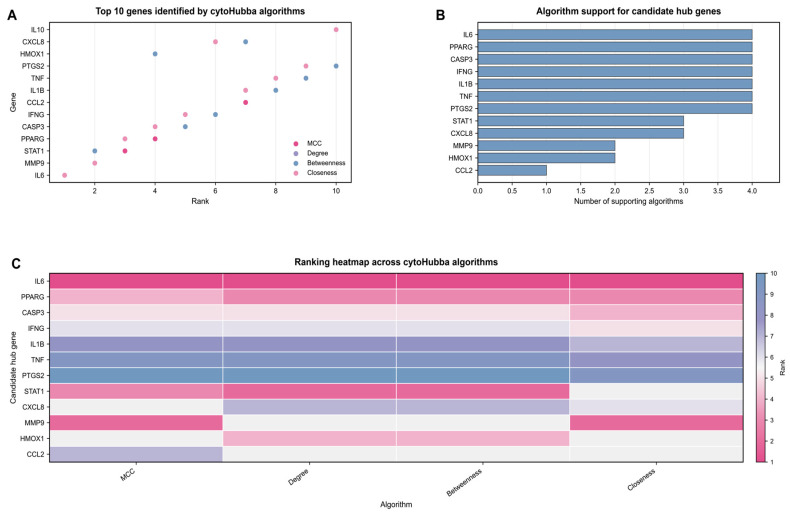
Multi-algorithm cytoHubba screening of candidate hub genes in the STRING 0.4 PPI network. (**A**) Distribution of genes ranked among the top 10 by the MCC, Degree, Betweenness, and Closeness algorithms. (**B**) Algorithm support for candidate hub genes, showing the number of algorithms in which each gene appeared among the top 10. (**C**) Ranking heatmap showing the relative ranking patterns of candidate hub genes across the four cytoHubba algorithms. Genes repeatedly ranked among the top 10 by multiple algorithms were considered to have higher network-topological priority.

Based on the PPI network constructed at the STRING interaction score threshold of 0.400, four cytoHubba algorithms, including MCC, Degree, Betweenness, and Closeness, were used to identify candidate hub genes. The top 10 genes ranked by each algorithm were compared. TNF, PTGS2, PPARG, IL6, IL1B, IFNG, and CASP3 were ranked among the top 10 genes by all four algorithms, indicating consistently high topological priority in the medium-confidence PPI network (Figure 3A,B). STAT1 and CXCL8 appeared among the top 10 genes in three algorithms, MMP9 and HMOX1 appeared in two algorithms, whereas IL10 and CCL2 appeared in one algorithm. The ranking heatmap showed that IL6, TNF, IL1B, PTGS2, PPARG, IFNG, and CASP3 maintained relatively stable rankings across different algorithms (Figure 3C). These results supported the preliminary prioritization of inflammation-, immunity-, apoptosis-, and tissue-injury-related candidate hub genes for subsequent cross-threshold assessment.

### 3.5. Cross-Threshold Integrated cytoHubba Screening and Stability Analysis

**Figure 4:**
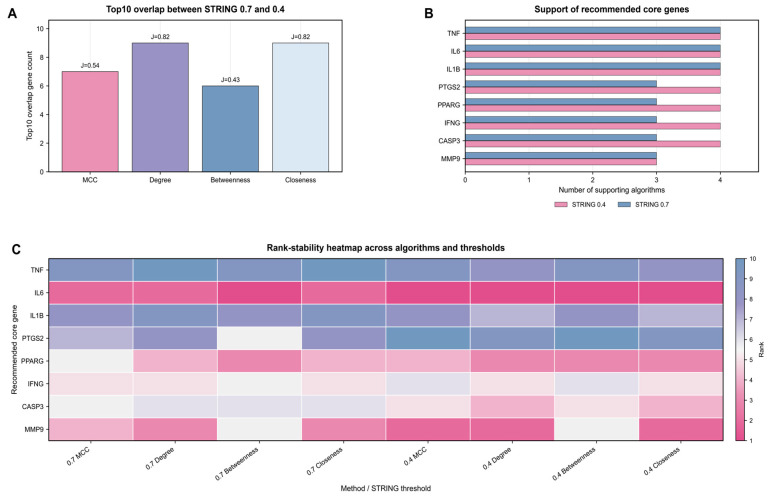
Cross-threshold assessment of prioritized core targets in the STRING 0.4 and STRING 0.7 PPI networks. (**A**) Overlap of the top 10 genes identified by MCC, Degree, Betweenness, and Closeness between the two STRING confidence thresholds. J indicates the Jaccard similarity index. (**B**) Algorithm support for the recommended core genes under the STRING 0.4 and STRING 0.7 thresholds. (**C**) Rank-stability heatmap showing the relative ranking patterns of the recommended core genes across algorithms and STRING thresholds. Genes with reproducible algorithm support and stable rankings across thresholds were retained as prioritized core targets.

To evaluate the robustness of hub-gene prioritization across different network-confidence conditions, the top 10 genes identified by MCC, Degree, Betweenness, and Closeness were compared between the STRING 0.4 and STRING 0.7 PPI networks. The numbers of overlapping top 10 genes between the two thresholds were 9 for Degree and Closeness, 7 for MCC, and 6 for Betweenness (Figure 4A), indicating that hub-gene prioritization was generally stable across the two STRING confidence thresholds.

TNF, IL6, and IL1B were ranked among the top 10 genes by all four algorithms under both STRING thresholds. PTGS2, PPARG, IFNG, and CASP3 were identified by all four algorithms in the STRING 0.4 network and by three algorithms in the STRING 0.7 network, whereas MMP9 was identified by three algorithms under both thresholds (Figure 4B). The rank-stability heatmap further showed that TNF, IL6, IL1B, PTGS2, PPARG, IFNG, CASP3, and MMP9 maintained relatively stable topological rankings across algorithms and thresholds (Figure 4C). Based on occurrence frequency, cross-threshold reproducibility, and ranking stability, these eight genes were retained as prioritized core targets for subsequent functional interpretation and molecular docking analysis. IL10 was considered an auxiliary candidate gene because its topological support was weaker and more variable across algorithms and thresholds.

### 3.6. MCODE Module Analysis and Module-Level Support for Candidate Core Targets

**Figure 5:**
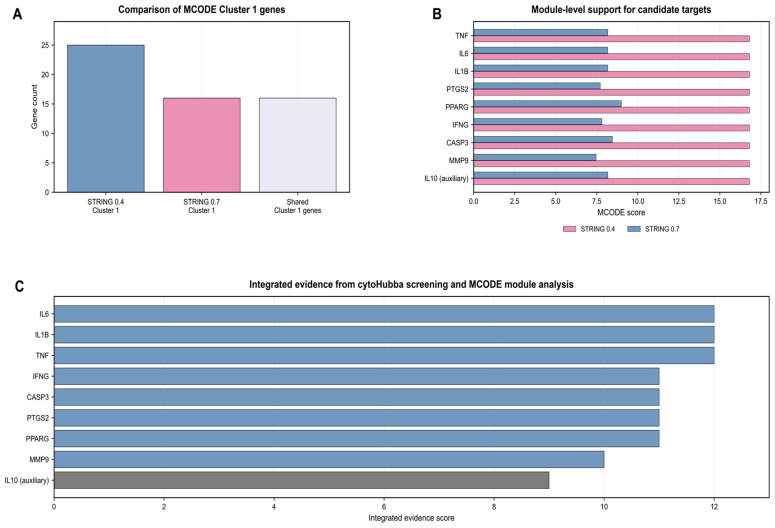
MCODE module analysis and integrated module-level support for candidate core targets. (**A**) Comparison of MCODE Cluster 1 gene counts between the STRING 0.4 and STRING 0.7 PPI networks. All 16 genes in the STRING 0.7 Cluster 1 were shared with the STRING 0.4 Cluster 1. (**B**) Module-level support for candidate targets under the two STRING confidence thresholds. (**C**) Integrated evidence score combining cytoHubba-based topological screening and MCODE module support. IL10 was retained as an auxiliary candidate gene rather than a prioritized core target.

To assess the candidate core targets at the network-module level, MCODE clustering analysis was performed on the STRING 0.4 and STRING 0.7 PPI networks. Cluster 1 contained 25 genes in the STRING 0.4 network and 16 genes in the STRING 0.7 network, and all 16 genes in the STRING 0.7 Cluster 1 were shared with the STRING 0.4 Cluster 1 (Figure 5A). This result indicated that the major densely connected module became more compact after increasing the STRING confidence threshold, while its core composition remained stable.

TNF, IL6, IL1B, PTGS2, PPARG, IFNG, CASP3, and MMP9 were all present in Cluster 1 under both STRING thresholds, indicating that these genes were consistently supported by the major network modules identified from the two PPI networks. IL10 was also included in Cluster 1 under both thresholds, suggesting module-level support, although its integrated evidence was weaker than that of the prioritized core targets (Figure 5B).

Integrated evidence from cytoHubba-based topological screening and MCODE module analysis showed that TNF, IL6, and IL1B had the strongest support, followed by PTGS2, PPARG, IFNG, CASP3, and MMP9. IL10 showed relatively weaker and less stable support and was therefore retained as an auxiliary candidate gene rather than a prioritized core target (Figure 5C). These findings suggest that the prioritized candidate targets potentially associated with the predicted host-directed effects of *H. cordata* in bovine *Mycoplasma bovis* pneumonia were mainly concentrated in stable inflammation- and immunity-related network modules.

### 3.7. GO Functional Enrichment Analysis

**Figure 6:**
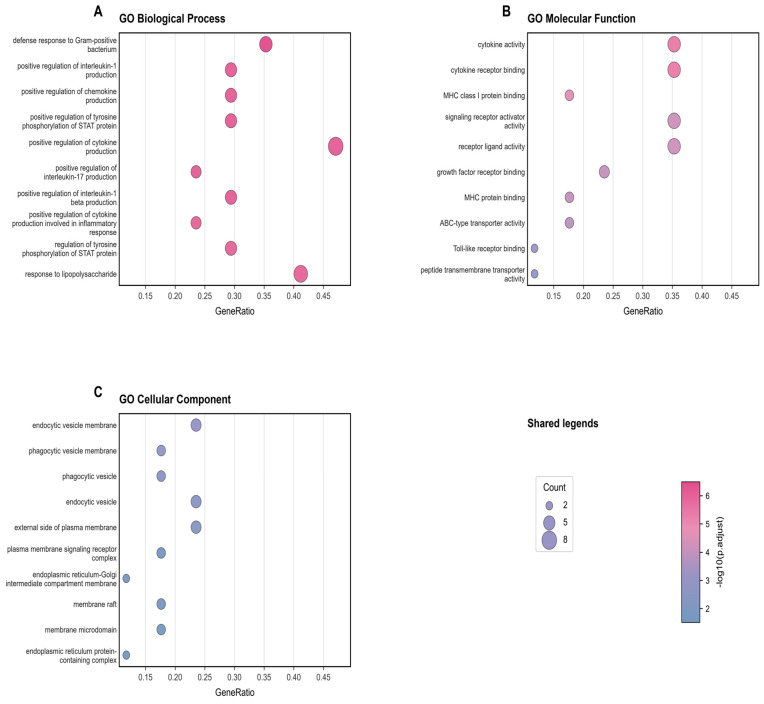
Gene Ontology (GO) enrichment analysis of common targets between *Houttuynia cordata* and bovine *Mycoplasma bovis* pneumonia. (**A**) Biological Process (BP) enrichment. (**B**) Molecular Function (MF) enrichment. (**C**) Cellular Component (CC) enrichment. Dot size represents the number of genes, and dot color represents −log10(adjusted *p* value).

GO enrichment analysis was performed on the common targets to clarify the biological functions potentially associated with the predicted host-directed effects of *Houttuynia cordata* in MBP. In the Biological Process (BP) category, the enriched terms were mainly related to defense response to Gram-positive bacterium, response to lipopolysaccharide, positive regulation of cytokine production, positive regulation of chemokine production, positive regulation of interleukin-1 production, positive regulation of interleukin-1 beta production, positive regulation of interleukin-17 production, and regulation of tyrosine phosphorylation of STAT protein (Figure 6A). These terms suggest that the common targets were primarily associated with bacterial stimulus-related host responses, cytokine and chemokine production, inflammatory amplification, and STAT-associated immune signaling.

In the Molecular Function (MF) category, the enriched terms mainly included cytokine activity, cytokine receptor binding, signaling receptor activator activity, receptor ligand activity, growth factor receptor binding, Toll-like receptor binding, MHC class I protein binding, MHC protein binding, ABC-type transporter activity, and peptide transmembrane transporter activity (Figure 6B). These results indicate that the common targets were mainly associated with cytokine–receptor interaction, receptor-mediated signaling, antigen-presentation-related molecular functions, and ligand–receptor regulatory activity.

In the Cellular Component (CC) category, significant terms were mainly related to membrane- and vesicle-associated structures, including endocytic vesicle membrane, phagocytic vesicle membrane, phagocytic vesicle, endocytic vesicle, external side of plasma membrane, plasma membrane signaling receptor complex, membrane raft, membrane microdomain, endoplasmic reticulum–Golgi intermediate compartment membrane, and endoplasmic reticulum protein-containing complex (Figure 6C). These CC terms were consistent with the involvement of common targets in membrane-associated receptor signaling, vesicle-related immune processes, and host-response regulation. GO enrichment analysis therefore supported the association of the common targets with inflammatory response, cytokine signaling, immune activation, bacterial stimulus response, and membrane-associated cellular processes.

### 3.8. KEGG Pathway Enrichment Analysis

**Figure 7:**
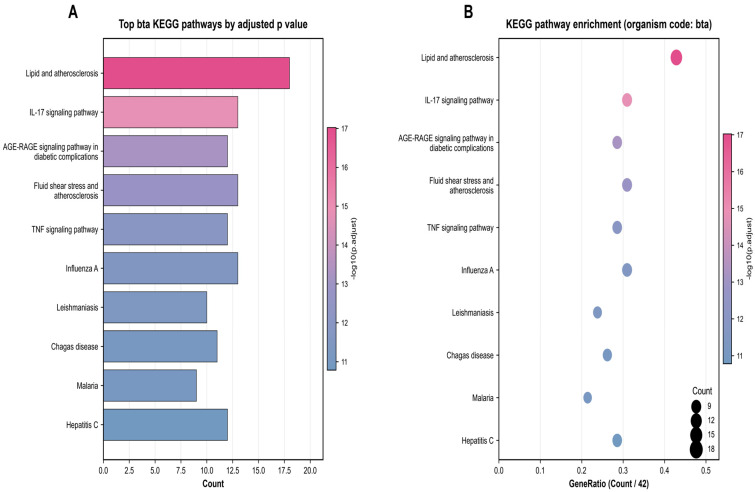
Kyoto Encyclopedia of Genes and Genomes (KEGG) pathway enrichment analysis of common targets between *Houttuynia cordata* and bovine *Mycoplasma bovis* pneumonia. The KEGG enrichment visualization was generated using *Bos taurus* mapping results with organism code bta. (**A**) Top KEGG pathways ranked by adjusted *p* value. (**B**) KEGG pathway enrichment dot plot. Dot size represents the number of genes, and dot color represents −log10(adjusted *p* value).

KEGG pathway enrichment analysis was performed to identify signaling pathways potentially associated with the predicted host-directed effects of Houttuynia cordata in MBP. The visualization was generated using Bos taurus mapping results with organism code bta. The common targets were enriched in multiple pathways related to inflammation, immune regulation, host stress responses, and infection-associated host-response signaling. The top enriched pathways displayed in Figure 7 included Lipid and atherosclerosis, IL-17 signaling pathway, AGE-RAGE signaling pathway in diabetic complications, Fluid shear stress and atherosclerosis, TNF signaling pathway, Influenza A, Leishmaniasis, Chagas disease, Malaria, and Hepatitis C (Figure 7A,B).

Among these pathways, the IL-17 and TNF signaling pathways were closely related to the inflammatory and immune-regulatory focus of this study. The IL-17 signaling pathway involved 13 genes, including PTGS2, TNF, CASP3, IKBKB, IL6, MAPK1, IFNG, CCL2, IL1B, CASP8, CXCL10, CXCL8, and MMP9. The TNF signaling pathway involved 12 genes, including PTGS2, TNF, SELE, CASP3, IKBKB, IL6, MAPK1, CCL2, IL1B, CASP8, CXCL10, and MMP9. These two pathways shared several inflammatory and injury-related genes, including TNF, IL6, IL1B, PTGS2, CASP3, CCL2, CXCL10, and MMP9, suggesting convergence on cytokine-mediated inflammatory signaling, apoptosis-related processes, chemokine-associated immune-cell recruitment, and tissue-injury responses.

Pathways named after specific diseases were interpreted as shared host-response or infection-associated signaling modules rather than direct evidence that H. cordata affects those diseases. The KEGG enrichment profile highlighted host inflammation–immune regulatory networks as an important pathway-level feature of the common targets between H. cordata and MBP.

### 3.9. Molecular Docking Analysis

Molecular docking analysis was performed to provide structural-level clues for the network pharmacology results by evaluating the predicted binding potential between representative compounds and prioritized targets. The docking results are summarized in Table 3. Except for 2-undecanone–PPARG, for which no stable binding conformation was observed under the present docking conditions, the other nine compound–target pairs showed binding energies lower than −5.0 kcal/mol.

Quercitrin showed the lowest predicted binding energy with PTGS2, with a binding energy of −7.8 kcal/mol. Quercetin showed favorable predicted binding with TNF and IL6, with binding energies of −7.4 and −7.1 kcal/mol, respectively. Quercitrin and kaempferol also showed favorable predicted binding with CASP3, with binding energies of −7.0 and −6.9 kcal/mol, respectively. Kaempferol–IL1B and quercetin–PPARG showed moderate predicted binding potential, with binding energies of −6.1 and −6.3 kcal/mol, respectively. Decanoyl acetaldehyde showed weaker predicted binding to MMP9 and PTGS2, both with binding energies of −5.5 kcal/mol.

**Figure 8:**
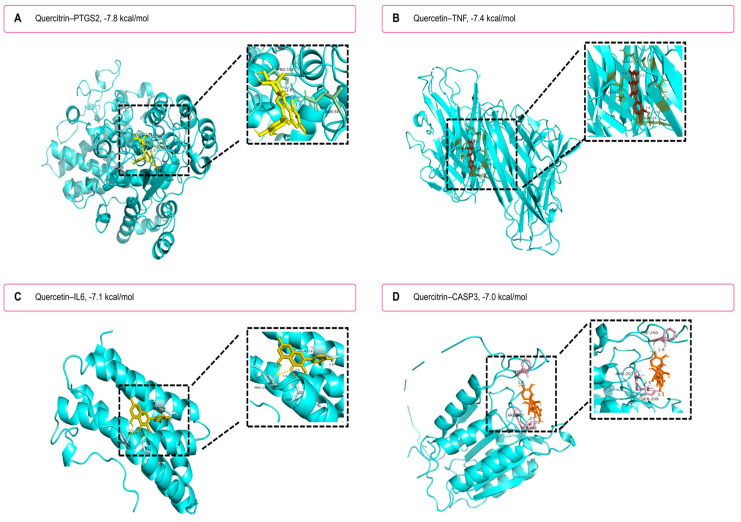
Representative molecular docking conformations of selected compound–target pairs. (**A**) Quercitrin–PTGS2, −7.8 kcal/mol. (**B**) Quercetin–TNF, −7.4 kcal/mol. (**C**) Quercetin–IL6, −7.1 kcal/mol. (**D**) Quercitrin–CASP3, −7.0 kcal/mol. Complete docking results for all tested ligand–target pairs are provided in Table 3. Cyan cartoon structures indicate receptor proteins, colored stick models indicate ligands, and dashed lines indicate hydrogen bonds or major non-covalent interactions.

Representative docking conformations of quercitrin–PTGS2, quercetin–TNF, quercetin–IL6, and quercitrin–CASP3 are shown in Figure 8. The docking profile suggested that flavonoid-related compounds in *H. cordata*, especially quercetin, quercitrin, and kaempferol, may be candidate ligands associated with inflammation-, immunity-, and apoptosis-related prioritized targets in MBP. These predicted ligand–target interactions require further validation in bovine-specific experimental models.

## 4. Discussion

This study used network pharmacology and molecular docking to explore potential host-directed mechanisms of *Houttuynia cordata* in the context o f bovine *Mycoplasma bovis* pneumonia (MBP). Integrated analysis based on PPI network construction, dual-threshold cytoHubba screening, MCODE module assessment, GO and KEGG enrichment analysis, and molecular docking prioritized TNF, IL6, IL1B, PTGS2, PPARG, IFNG, CASP3, and MMP9 as candidate core targets. These targets were mainly associated with cytokine-mediated inflammatory amplification, immune regulation, apoptosis, oxidative-stress-related processes, and tissue-injury responses. Enrichment of IL-17 and TNF signaling pathways further suggested that the predicted host-directed relevance of *H. cordata* was more closely related to host inflammatory and immune-response networks than to direct elimination of *M. bovis*. Molecular docking provided structural-level clues for predicted interactions between representative flavonoid-related compounds and inflammation- or apoptosis-associated targets, especially quercitrin–PTGS2, quercetin–TNF, quercetin–IL6, and quercitrin–CASP3. These findings should be interpreted as computationally derived mechanistic hypotheses for future bovine-specific experimental assessment.

The difficulty in controlling MBP should be considered not only in relation to pathogen persistence, but also in relation to the host pathological network involving inflammatory amplification, immune dysregulation, apoptosis, and pulmonary tissue injury. Previous studies have reported that *M. bovis* infection is associated with immune dysfunction, persistent infection, and host–cell injury-related responses [34,35]. In bovine host–cell and tissue studies, *M. bovis* infection has been linked to inflammatory responses involving cytokines and immune-related mediators such as IL1B, IL6, TNF, CXCL8, and NF-κB-related signaling components [35,36,37]. Consistent with this pathological background, TNF, IL6, and IL1B were identified as stable candidate core targets in the present analysis and were involved in GO terms related to positive regulation of cytokine production, chemokine production, interleukin-1 production, interleukin-17 production, and bacterial stimulus-related host responses. These targets were also involved in KEGG pathways related to IL-17 and TNF signaling, suggesting that the common targets may reflect cytokine-centered inflammatory regulation in the MBP context. PTGS2 may further connect inflammatory mediator production with immune dysregulation, because prostaglandin E2, a downstream product of the COX-2/PTGS2 axis, has been implicated in PD-1/PD-L1-mediated immune impairment during *M. bovis* infection [34]. PPARG may represent an additional immunometabolic regulatory node, as PPARγ has been associated with macrophage inflammatory regulation and pulmonary inflammation control after respiratory infection [38,39]. The identification of CASP3 and MMP9 further extended the predicted target profile from inflammatory regulation to cell injury, apoptosis, matrix remodeling, and pulmonary tissue damage. This interpretation is consistent with the established role of caspases, including caspase-3, in apoptosis-related substrate cleavage and the involvement of MMP9-related matrix metalloproteinase activity in acute lung injury, tissue remodeling, and repair responses [40,41].

At the compound level, the predicted host-directed relevance of *H. cordata* in MBP may be associated with multiple classes of constituents, including flavonoids, volatile components, polysaccharides, alkaloids, and organic acids. Phytochemical and metabolomic studies have reported diverse bioactive constituents in *H. cordata* extracts and have highlighted flavonoid-related and volatile components as important chemical features [42,43]. Flavonoids such as quercetin, quercitrin, and kaempferol have been frequently described as bioactive compounds with inflammation- and oxidative-stress-related relevance, whereas volatile constituents such as decanoyl acetaldehyde and 2-undecanone contribute to the characteristic chemical profile and source specificity of *H. cordata* [42,43,44]. Quercetin has also been discussed as a bioactive feed additive in poultry and rabbits, with reported relevance to antioxidant status, intestinal health, immune function, and animal-derived product quality [45]. More broadly, phytogenic feed additives have been evaluated in livestock production for their effects on growth performance and immune response, supporting their potential role as complementary resources in animal health management rather than as direct antimicrobial substitutes [46]. Although these animal studies cannot be directly extrapolated to bovine MBP, they provide useful comparative evidence supporting the broader animal-health relevance of flavonoid-related and phytogenic compounds. In the present molecular docking analysis, relatively lower binding energies were mainly observed for flavonoid–target pairs, including quercitrin–PTGS2, quercetin–TNF, quercetin–IL6, quercitrin–CASP3, and kaempferol–CASP3. These results suggest that flavonoids may provide stronger structural-level clues for predicted interactions with inflammation- and apoptosis-related targets, especially those involved in TNF/IL6-mediated cytokine signaling, PTGS2-related inflammatory mediator production, and CASP3-associated apoptosis. By contrast, decanoyl acetaldehyde showed weaker predicted binding with MMP9 and PTGS2, and no stable binding conformation was observed between 2-undecanone and PPARG under the present docking conditions. Therefore, volatile constituents may retain pharmacological and source-specific relevance, but their direct predicted binding potential with the selected protein targets appeared weaker than that of flavonoids in this docking model. These findings suggest that the compound-level basis of *H. cordata* in the MBP context should not be attributed to a single constituent class. Flavonoids may represent the main structural contributors to the predicted ligand–target interactions identified in this study, whereas volatile constituents, polysaccharides, and other components may participate through auxiliary, complementary, or indirect regulatory effects.

At the pathway level, enrichment of the IL-17 and TNF signaling pathways extended the interpretation of *H. cordata* from individual candidate targets to host inflammation–immunity networks in the MBP context. The IL-17 pathway is involved in mucosal immunity and inflammatory responses, contributing to host defense against extracellular pathogens while also potentially amplifying inflammatory injury under persistent or excessive immune activation [47]. In *M. bovis* infection-related studies, virulent strains have been associated with enhanced Th17 inflammatory responses, and transcriptomic analyses of infected calves have reported airway inflammatory injury accompanied by immune- and inflammation-related transcriptional alterations [36,37]. Consistent with these findings, the IL-17 signaling pathway in the present analysis involved PTGS2, TNF, CASP3, IL6, IFNG, IL1B, CXCL8, and MMP9, suggesting that this pathway may link cytokine release, inflammatory-cell recruitment, apoptosis, and tissue remodeling. The TNF signaling pathway showed a similar inflammatory network pattern, involving cytokine- and chemokine-related targets such as TNF, IL6, IL1B, CCL2, CXCL10, and MMP9. This pathway-level convergence suggests that the predicted host-directed relevance of *H. cordata* may be associated with infection-induced inflammatory cascades, chemokine-mediated immune-cell recruitment, apoptosis-related processes, and tissue-injury responses rather than with a single molecular node. Several enriched KEGG pathways with disease names, such as Influenza A, Leishmaniasis, Chagas disease, Malaria, Hepatitis C, AGE-RAGE signaling pathway in diabetic complications, and Fluid shear stress and atherosclerosis, should not be interpreted as direct evidence that *H. cordata* affects these diseases. Because KEGG pathway maps represent molecular interaction and reaction networks, these disease-name pathways are more appropriately interpreted as shared inflammatory, immune, oxidative-stress-related, and tissue-injury-related molecular modules [48]. This pathway profile indicates that the common targets between *H. cordata* and MBP mainly reflect host inflammatory response, immune regulation, oxidative-stress-related processes, and tissue-injury networks rather than a specific non-MBP disease context.

This study remains a computational prediction study based mainly on network pharmacology and molecular docking; therefore, the results should be interpreted as candidate mechanistic clues rather than direct functional evidence. Disease-related targets were collected from GeneCards, OMIM, and CTD, which are largely based on human-centered or general disease knowledge. Although the species was restricted to *Bos taurus* during PPI analysis, and dual-confidence STRING networks, multi-algorithm cytoHubba screening, and MCODE module assessment were used to improve the robustness of core-target prioritization, cross-species mapping bias and database annotation limitations remain unavoidable. In addition, molecular docking only provides structural-level clues for potential ligand–target interactions and does not demonstrate that *H. cordata* or its individual constituents can regulate these targets in bovine cells or animals. The pharmacokinetic behavior, tissue distribution, effective exposure levels, dose–response relationships, and safety profile of *H. cordata* constituents in cattle were also not evaluated in this study.

Future studies should use bovine-derived experimental systems, such as primary bovine alveolar macrophages, bovine monocyte-derived macrophages, or bovine respiratory epithelial cells, to examine key targets including TNF, IL6, IL1B, PTGS2, PPARG, IFNG, CASP3, and MMP9. qPCR could be used for transcript-level assessment, whereas ELISA, Western blotting, immunofluorescence, or related assays could be used for protein-level and pathway-level assessment. Representative compounds such as quercetin, quercitrin, and kaempferol should also be evaluated for their effects on inflammatory mediator release, apoptosis-related indicators, oxidative-stress-related responses, and tissue-injury-associated markers. From an application perspective, *H. cordata* should be positioned as a candidate medicinal-edible auxiliary resource for supporting host inflammation–immune regulation and respiratory health management under antimicrobial-reduction strategies, rather than as a direct substitute for veterinary diagnosis or antimicrobial therapy against *M. bovis*. This study provides systematic mechanistic clues and candidate compound–target pairs for future bovine-specific experimental assessment of *H. cordata* in the MBP context.

**Figure 9:**
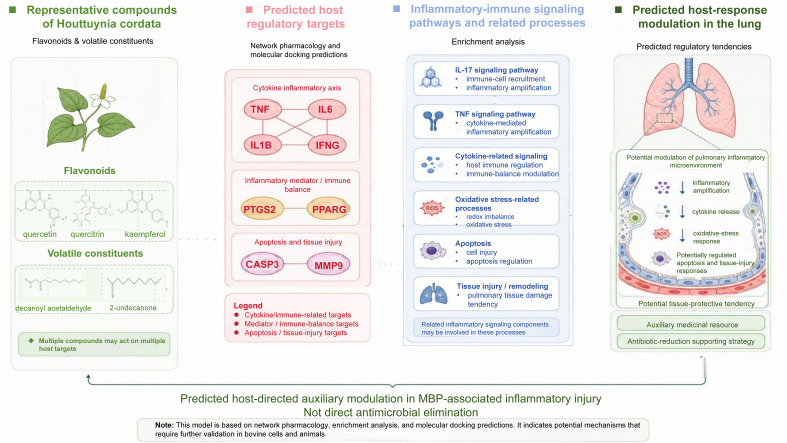
Proposed host-directed mechanism by which *Houttuynia cordata* may modulate inflammation- and immunity-related injury in bovine *Mycoplasma bovis* pneumonia. Representative compounds of *H. cordata*, especially flavonoids such as quercetin, quercitrin, and kaempferol, may interact with predicted host regulatory targets including TNF, IL6, IL1B, PTGS2, PPARG, IFNG, CASP3, and MMP9. These targets are mainly associated with IL-17 signaling, TNF signaling, cytokine-mediated inflammatory amplification, oxidative-stress-related processes, apoptosis, and tissue-injury-related responses. This model suggests that *H. cordata* may act as a host-directed auxiliary medicinal-edible resource for modulating MBP-associated inflammatory and immune disturbances, rather than as a direct antimicrobial substitute. Arrows indicate the proposed direction of computationally inferred associations from representative compounds to predicted host targets, enriched inflammatory and immune pathways, and potential host-response modulation.

Based on these integrated findings, the proposed host-directed mechanism by which *Houttuynia cordata* may modulate inflammation- and immunity-related injury in bovine Mycoplasma bovis pneumonia is summarized in Figure 9.

## 5. Conclusions

This network pharmacology and molecular docking study suggests that *Houttuynia cordata* may modulate bovine *Mycoplasma bovis* pneumonia (MBP)-associated pathological processes mainly through host-directed regulation of inflammation- and immunity-related networks. Integrated network analysis identified TNF, IL6, IL1B, PTGS2, PPARG, IFNG, CASP3, and MMP9 as key candidate targets, indicating that the predicted effects of *H. cordata* may involve inflammatory amplification, cytokine signaling, immune regulation, apoptosis, oxidative stress, and tissue-injury-related processes. Molecular docking further suggested that flavonoid compounds, particularly quercitrin and quercetin, may provide an important structural basis for interactions with inflammation- and apoptosis-related targets. Rather than serving as a direct antimicrobial substitute, *H. cordata* may be more appropriately positioned as a medicinal and edible auxiliary resource for supporting host inflammation–immune regulation and respiratory health management under antimicrobial-reduction strategies. These findings provide candidate compounds, targets, and mechanistic clues for future bovine-specific experimental validation.

## Figures and Tables

**Table 1:** Representative compounds of *Houttuynia cordata* selected for network interpretation and molecular docking.

Compound	Category	OB (%)	DL	Selection Source and Rationale	Role in This Study
Quercetin	Flavonoid	46.43	0.28	TCMSP and literature support	Docking ligand; inflammation-related predicted target association
Kaempferol	Flavonoid	41.88	0.24	TCMSP and literature support	Docking ligand; apoptosis- and inflammation-related predicted target association
Quercitrin	Flavonoid glycoside	34.04	0.74	Database and literature support	Docking ligand; PTGS2- and CASP3-related predicted interaction
Decanoyl acetaldehyde	Volatile constituent	36.04	0.04	TCMSP and literature support	Characteristic volatile component
2-Undecanone	Volatile constituent	17.66	0.03	TCMSP and literature support	Characteristic volatile component

Note: OB, oral bioavailability; DL, drug-likeness. OB ≥ 30% and DL ≥ 0.18 were used as preliminary TCMSP screening criteria. Quercetin, kaempferol, and quercitrin met the preliminary ADME screening criteria and were retained as representative flavonoid-related compounds. Decanoyl acetaldehyde and 2-undecanone were retained as characteristic volatile constituents of *H. cordata* based on literature support and source specificity, although they did not fully meet the conventional TCMSP ADME thresholds. Therefore, these volatile constituents were interpreted as literature-curated representative constituents rather than strictly ADME-filtered compounds.

**Table 2:** Functional classification of representative common targets between *Houttuynia cordata* and bovine *Mycoplasma bovis* pneumonia.

Functional Category	Representative Genes
Cytokines and cytokine signaling	TNF, IL6, IL1B, IL1A, IFNG, IL10
Chemokines and immune-cell recruitment	CCL2, CXCL8, CXCL10
Inflammatory signaling	IKBKB, STAT1, MAPK1, PTGS2
Oxidative stress and redox regulation	NOS2, NOS3, SOD1, HMOX1, GSTM1, NCF1
Apoptosis and cell injury	CASP3, CASP8, TP53, PARP1
Tissue remodeling and injury	MMP9, PLAU, F3, SLPI
Metabolic and transcriptional regulation	PPARG, RXRA, EGFR, MYC, RUNX2, DPP4

**Note:** The 43 common targets were classified according to their known biological functions and their relevance to MBP-associated pathological processes, including cytokine signaling, chemokine-mediated immune-cell recruitment, inflammatory signal transduction, oxidative stress and redox regulation, apoptosis, tissue injury, and metabolic or transcriptional regulation. The genes listed in the table are representative examples for each functional category. Some genes may participate in more than one biological process; therefore, the functional categories are used for mechanistic interpretation rather than strict mutually exclusive classification.

**Table 3:** Molecular docking binding energies between representative compounds and prioritized targets.

Compound	Target	Binding Energy (kcal/mol)	Interpretation
Quercitrin	PTGS2	−7.8	Favorable predicted binding
Quercetin	TNF	−7.4	Favorable predicted binding
Quercetin	IL6	−7.1	Favorable predicted binding
Quercitrin	CASP3	−7.0	Favorable predicted binding
Kaempferol	CASP3	−6.9	Favorable predicted binding
Quercetin	PPARG	−6.3	Moderate predicted binding
Kaempferol	IL1B	−6.1	Moderate predicted binding
Decanoyl acetaldehyde	MMP9	−5.5	Weak-to-moderate predicted binding
Decanoyl acetaldehyde	PTGS2	−5.5	Weak-to-moderate predicted binding
2-Undecanone	PPARG	No stable conformation	Limited predicted binding potential

**Note:** Binding energy < −5.0 kcal/mol was considered to indicate possible binding potential under the present docking conditions. Molecular docking provides structural-level clues but does not directly prove biological regulation or functional activity.

## Data Availability

The original contributions presented in this study are included in the article/Supplementary Materials. Further inquiries can be directed to the corresponding author.

## Supplementary Materials

The following supporting information can be downloaded at: https://www.mdpi.com/article/10.3390/vetsci13070658/s1, Table S1: Integrated evidence matrix for prioritized core targets; Table S2: Ligand information, receptor structures, docking-region definitions, and docking results used in molecular docking analysis.
