# Supplementary material for: Potential Host-Directed Mechanisms of Houttuynia cordata in Bovine Mycoplasma bovis Pneumonia: A Network Pharmacology and Molecular Docking Study
Source: Vet Sci. 2026 Jul 7;13(7):658. doi: 10.3390/vetsci13070658 (PMC13418644; doi:10.3390/vetsci13070658)
Supplement: Supplementary file 1 [file vetsci-13-00658-s001.zip › vetsci-4374672-supplementary.pdf]

Supplementary Table S1. Ligand information, receptor structures, docking-region definitions, and docking results used in molecular docking analysis.

No.	Ligand	PubChem CID	Target	PDB ID	Structure-selection rationale	Docking-region definition	Binding energy (kcal/mol)
1	Quercitrin	5280459	PTGS2	5IKQ	PTGS2/COX-2 structure containing a co-crystallized inhibitor-binding region; suitable for evaluating ligand interaction with the COX-2 inflammatory mediator target.	Docking region was defined around the co-crystallized ligand-binding pocket of PTGS2/COX-2.	−7.8
2	Quercetin	5280343	TNF	2AZ5	TNF-α structure with a small-molecule binding/interface-associated region; suitable for evaluating potential interaction with a cytokine-related target.	Docking region was defined around the reported small-molecule binding or interface-associated region of TNF.	−7.4
3	Quercetin	5280343	IL6	1ALU	IL-6 crystal structure; selected to evaluate potential interaction with a cytokine-related target lacking a conventional enzyme-like active pocket.	Docking region was defined around a reported functional or interface-associated region of IL6.	−7.1
4	Quercitrin	5280459	CASP3	1NMS	Caspase-3 structure containing an inhibitor-associated catalytic region; suitable for evaluating interactions related to apoptosis-associated target regulation.	Docking region was defined around the inhibitor-associated catalytic pocket of CASP3.	−7.0
5	Kaempferol	5280863	CASP3	1NMS	Caspase-3 structure containing an inhibitor-associated catalytic region; suitable for evaluating interactions related to apoptosis-associated target regulation.	Docking region was defined around the inhibitor-associated catalytic pocket of CASP3.	−6.9

6	Quercetin	5280343	PPARG	3V9V	PPARγ ligand-binding domain structure; suitable for evaluating ligand interaction with an immune-metabolic regulatory target.	Docking region was defined around the ligand-binding pocket of the PPARG ligand-binding domain.	−6.3
7	Kaempferol	5280863	IL1B	1ITB	IL-1β structure; selected to evaluate potential interaction with a cytokine-associated functional or interface-associated region.	Docking region was defined around the IL1B functional or interface-associated region after receptor-chain processing, if applicable.	−6.1
8	Decanoyl acetaldehyde	122640	MMP9	1GKC	MMP9 catalytic-domain structure containing an inhibitor-associated region; suitable for evaluating interaction with a tissue-remodeling-related target.	Docking region was defined around the inhibitor-associated catalytic pocket of MMP9.	−5.5
9	Decanoyl acetaldehyde	122640	PTGS2	5IKQ	PTGS2/COX-2 structure containing a co-crystallized inhibitor-binding region; suitable for evaluating ligand interaction with the COX-2 inflammatory mediator target.	Docking region was defined around the co-crystallized ligand-binding pocket of PTGS2/COX-2.	−5.5
10	2-Undecanone	8163	PPARG	3V9V	PPARγ ligand-binding domain structure; suitable for evaluating ligand interaction with an immune-metabolic regulatory target.	Docking region was defined around the ligand-binding pocket of the PPARG ligand-binding domain.	No stable conformation

Note: CID, PubChem compound identifier; PDB, Protein Data Bank; PTGS2, prostaglandin-endoperoxide synthase 2; TNF, tumor necrosis factor; IL6, interleukin 6; CASP3, caspase 3; PPARG, peroxisome proliferator-activated receptor gamma; IL1B, interleukin 1 beta; MMP9, matrix metallopeptidase 9. Receptor structures were obtained from the RCSB PDB database. Docking regions were defined according to co-crystallized ligand-binding pockets, known functional pockets, or reported functional/interface-associated regions. Binding energy < -5.0 kcal/mol was used as an empirical indicator of possible binding potential under

the present docking conditions. Molecular docking provides structural-level clues for potential ligand–target interactions but does not directly prove biological regulation or functional activity.

Supplementary Table S2. Integrated evidence matrix for prioritized core targets.

Target	Functional interpretation	Network-topological evidence	MCODE module evidence	GO-related evidence	KEGG-related evidence	Docking-related evidence	Overall interpretation
TNF	Central cytokine-related inflammatory node.	Ranked among the top 10 genes by MCC, Degree, Betweenness, and Closeness under both STRING 0.4 and STRING 0.7 thresholds.	Present in MCODE Cluster 1 under both STRING thresholds.	Associated with cytokine production, chemokine production, bacterial stimulus response, and inflammatory amplification.	Included in IL-17 signaling and TNF signaling pathways.	Quercetin–TNF showed favorable predicted binding energy (−7.4 kcal/mol).	High-priority candidate target reflecting cytokine-centered host inflammatory regulation.
IL6	Pro-inflammatory cytokine and immune-response mediator.	Ranked among the top 10 genes by all four cytoHubba algorithms under both STRING thresholds.	Present in MCODE Cluster 1 under both STRING thresholds.	Associated with cytokine production, inflammatory response, and immune activation.	Included in IL-17 signaling and TNF signaling pathways.	Quercetin–IL6 showed favorable predicted binding energy (−7.1 kcal/mol).	High-priority candidate target related to inflammatory amplification and immune-response modulation.
IL1B	Inflammatory cytokine related to innate immune activation.	Ranked among the top 10 genes by all four cytoHubba algorithms under both STRING thresholds.	Present in MCODE Cluster 1 under both STRING thresholds.	Associated with interleukin-1 production, interleukin-1 beta production, cytokine production, and bacterial stimulus response.	Included in IL-17 signaling and TNF signaling pathways.	Kaempferol–IL1B showed moderate predicted binding energy (−6.1 kcal/mol).	High-priority candidate target related to innate inflammation and cytokine-network regulation.
PTGS2	Inflammatory mediator-related target associated with COX-2/prostaglandin signaling.	Ranked among the top 10 genes by all four algorithms in the STRING 0.4 network and by three algorithms in the STRING 0.7 network.	Present in MCODE Cluster 1 under both STRING thresholds.	Associated with inflammatory response, cytokine-related processes, and receptor-mediated host-response regulation.	Included in IL-17 signaling and TNF signaling pathways.	Quercitrin–PTGS2 showed the lowest predicted binding energy (−7.8 kcal/mol); decanoyl acetaldehyde–PTGS2 showed weaker predicted binding (−5.5 kcal/mol).	Prioritized inflammatory mediator node with strong docking-level clues for flavonoid-related interaction.

Target	Functional interpretation	Network-topological evidence	MCODE module evidence	GO-related evidence	KEGG-related evidence	Docking-related evidence	Overall interpretation
PPARG	Immunometabolic regulatory target related to macrophage inflammatory regulation.	Ranked among the top 10 genes by all four algorithms in the STRING 0.4 network and by three algorithms in the STRING 0.7 network.	Present in MCODE Cluster 1 under both STRING thresholds.	Associated with metabolic and transcriptional regulation and immune-related host-response interpretation.	Linked to host inflammatory and immune-regulatory pathway interpretation rather than a single disease-specific pathway.	Quercetin–PPARG showed moderate predicted binding energy (−6.3 kcal/mol); 2-undecanone–PPARG showed no stable conformation.	Candidate immunometabolic regulatory node that may connect inflammation and host-response modulation.
IFNG	Th1-related cytokine and immune-regulatory mediator.	Ranked among the top 10 genes by all four algorithms in the STRING 0.4 network and by three algorithms in the STRING 0.7 network.	Present in MCODE Cluster 1 under both STRING thresholds.	Associated with immune activation, cytokine signaling, and host defense-related interpretation.	Included in IL-17 signaling and infection-associated host-response pathway interpretation.	Not selected for molecular docking in the present representative ligand–target set.	Candidate immune-regulatory target supported mainly by network topology, module membership, and enrichment evidence.
CASP3	Apoptosis-associated target related to cell injury.	Ranked among the top 10 genes by all four algorithms in the STRING 0.4 network and by three algorithms in the STRING 0.7 network.	Present in MCODE Cluster 1 under both STRING thresholds.	Associated with apoptosis, cell injury, and tissue-injury-related processes.	Included in IL-17 signaling and TNF signaling pathways.	Quercitrin–CASP3 and kaempferol–CASP3 showed favorable predicted binding energies (−7.0 and −6.9 kcal/mol, respectively).	Candidate apoptosis-related target connecting inflammatory signaling with host-cell injury.
MMP9	Tissue remodeling and injury-related matrix metalloproteinase.	Ranked among the top 10 genes by three algorithms under both STRING 0.4 and STRING 0.7 thresholds.	Present in MCODE Cluster 1 under both STRING thresholds.	Associated with tissue injury, extracellular remodeling, chemokine-related inflammatory responses, and host-response regulation.	Included in IL-17 signaling and TNF signaling pathways.	Decanoyl acetaldehyde–MMP9 showed weak to moderate predicted binding energy (−5.5 kcal/mol).	Candidate tissue-injury/remodeling target supported by cross-threshold topology and pathway-level evidence.

Note: STRING 0.4 and STRING 0.7 indicate medium- and high-confidence STRING interaction thresholds, respectively. The matrix integrates common-target status, cytoHubba-based topological support, MCODE module membership, GO and KEGG enrichment evidence, docking results, and mechanistic interpretation. This integrated evidence matrix is descriptive and hypothesis-generating; it does not represent experimental validation. IL10 was retained as an auxiliary candidate gene in

the main manuscript and was not included among the prioritized core targets listed in this table.
